# Generating keV ion distributions for nuclear reactions at near solid-density using intense short-pulse lasers

**DOI:** 10.1038/s41467-019-12076-x

**Published:** 2019-09-13

**Authors:** A. J. Kemp, S. C. Wilks, E. P. Hartouni, G. Grim

**Affiliations:** Lawrence Livermore National Laboratory, Livermore, CA CA94550 USA

**Keywords:** Nuclear physics, Laser-produced plasmas

## Abstract

Our understanding of a large range of astrophysical phenomena depends on a precise knowledge of charged particle nuclear reactions that occur at very low rates, which are difficult to measure under relevant plasma conditions. Here, we describe a method for generating dense plasmas at effective ion temperatures >20 keV, sufficient to induce measurable charged particle nuclear reactions. Our approach uses ultra-intense lasers to drive micron-sized, encapsulated nanofoam targets. Energetic electrons generated in the intense laser interaction pass through the foam, inducing a rapid expansion of the foam ions; this results in a hot, near-solid density plasma. We present the laser and target conditions necessary to achieve these conditions and illustrate the system performance using three-dimensional particle-in-cell simulations, outline potential applications and calculate expected nuclear reaction rates in the D(d,n) and ^12^C(p,γ) systems assuming CD, or CH aerogel foams.

## Abstract (web summary)

Studying nuclear reactions in an astrophysical plasma environment is challenging but laboratory experiments can mimic such extreme conditions. Here the authors discuss the potential use of intense laser-produced dense plasma to find the rates of nuclear reactions in plasma-screened conditions.

## Introduction

Our understanding of a large range of astrophysical phenomena depends on a precise knowledge of charged particle nuclear reactions that occur at very low rates^1–9^. Historically, measuring these reactions in astrophysically relevant conditions has not been possible; accelerator based measurements have been utilized, leaving open the question of whether screening corrections from a cold solid density target to a plasma are correct. Furthermore, accelerator measurements at low center-of-mass energies suffer from low reaction rates due to low beam currents, substantial Coulomb barrier suppression, and backgrounds that in some cases force experiments deep underground^10^. In the present work, we discuss a platform that would provide a microscopic volume of near-solid density plasma with an ion temperature of tens of millions of degrees, where measurable reaction products would be created at conditions similar to those found in astrophysical objects like stars.

Previous experiments with short-pulse lasers^11–15^ have directly irradiated highly aligned nanowires. While that approach maximizes laser absorption and energy of accelerated ions, it creates large and complicated gradients in the ion spatial and velocity distribution functions. Similarly, recent experiments with ultra-intense laser light directly irradiating low-density (<160 mg/cc) foam^16^ were shown to yield multi-MeV ion spectra. This lack of control over plasma conditions makes this method problematic for use in nuclear reaction measurements, where these quantities must be relatively isotropic. Coulomb exploded clusters using intense, ultrafast lasers have also been used to generate nuclear reactions^17–21^ relevant to astrophysics, but suffer from similar gradient issues.

**Fig. 1:**
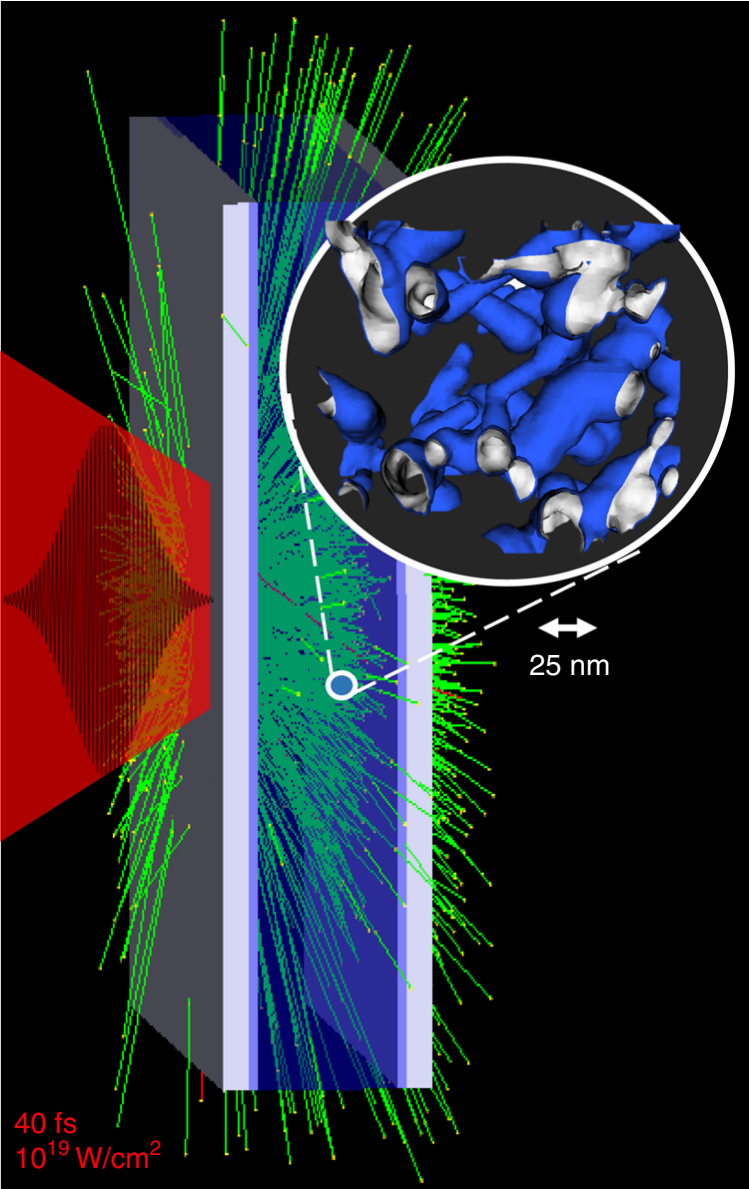
Schematic setup of encapsulated nanofoam target. A laser pulse (40 fs long, 10^19^ W/cm^2^, indicated in red) generates relativistic electrons that flood the target, causing quasi-static electric fields that expand the unstructured foam (foam structure is shown in inset; the typical scale of the foam is 25 nm)

In the following, we propose to use the TNSA mechanism to induce rapid expansion of near-solid density, unstructured nanofoam targets protected from direct laser irradiation by a solid layer, as shown in Fig. 1^22^. The dominant process by which ions are accelerated in the interaction of an intense sub-picosecond laser pulse with solid matter is target-normal sheath acceleration (TNSA)^23^. Laser-generated relativistic electrons create electrostatic Debye sheath fields directed normal to target surfaces, lasting several times the laser pulse duration; the amplitude of these fields is of the order of TV/m and scales directly with laser intensity; in experiments where intense short-pulse laser pulses irradiate solid micrometer thin foils, TNSA has been found to accelerate ions to tens of MeV/u^24^.

Flooding the target with MeV electrons rapidly ionizes and accelerates ions normal to the surfaces of nanometer scale filaments that make up the foam, turning it into a uniform, isotropic plasma within hundreds of femtoseconds. We present a computational study of this mechanism by means of collisional particle-in-cell (PIC) simulations combined with arguments on how these targets scale. We then compute nuclear reaction rates based on the kinematics of individual reactions, where the actual ion energy distribution is obtained from the PIC simulation results. Initial experiments could use materials with well-known cross sections and reaction products would be collected to diagnose plasma conditions; once the plasma conditions are known, one could detect the products of lesser-known nuclear reactions to determine their cross sections. Our system allows the study of nuclear cross sections that would be inaccessible to accelerator studies, as the reactant density is ten orders of magnitude higher than in an accelerator. Furthermore, this platform enables access to measurements of reaction rates in plasma conditions, unlike the cold target measurements made using accelerators.

## Results

### Laser-driven nanofoam targets

As diagrammed in Fig. 1, we focus on a scenario where a 40 fs-fwhm, 3 × 10^19^ W/cm^2^ laser pulse at 1 micrometer wavelength impinges a solid-density foil abutting a nanoscale structured plastic foam. As illustrated in the figure, and included in the simulation results, a second downstream abutting foil, was included, though this layer plays no role in effecting the ion distribution functions. To illustrate the principle qualitatively, we have performed a one-dimensional particle in cell simulation of this setup, where the foam region is represented by a 50 nm-period rectangular function density profile with a 50% fill factor of solid density CD plastic at *ρ* = 1.1 g/cc. A pre-plasma scale length of 0.3 µm was chosen based on a 1:1 mix of C^+6^ and D^+^ ions, giving an electron density, *n*_*e*_ = 350 *n*_*c*_, where $n_c = m_e\omega _L^2/4\pi e^2,$is the critical density for 1 μm wavelength light. This choice was guided by previous work simulating the interaction of an intrinsic ASE pre-pulse that typically precedes drive pulses in high power, short-pulse laser systems. Our PIC simulations were performed using the collisional particle-in-cell code PSC^25^, see Section “Methods”, at a resolution of 480 cells per micrometer, corresponding to five cells per collisionless skin depth $l_s = \frac{c}{{\omega _p}}$, or ~10 nm. Each cell contained 14,400 electron and ion particles. The physical size of the simulated volume was 20 μm. Fiducial simulations at five times higher spatial resolution, corresponding to a cell size of the electron Debye length at solid density and *T*_e_ = 900 eV, as well as simulations at one-half times the nominal resolution, agree with our first results, suggesting numerical convergence. It is worth noting that the electron Debye length of this system, $l_D = \frac{{v_{{\mathrm{th}}}}}{{\omega _p}}$, is ~0.1 nm at solid density in a plasma with *T*_*e*_ < 100 eV. This is smaller than both the thickness of our foam ligaments, as well as the interatomic distances at solid density, ${\mathrm{n}}_i^{ - 1/3}\sim 0.4\,{\mathrm{nm}}$, which means that the dynamics result from cold ions at solid density rapidly expanding into vacuum^26,27^, rather than being driven via Coulomb explosion^17,28^. Further, in a Coulomb explosion, ions and electrons are completely separated, and the notion of a plasma does not apply.

**Fig. 2:**
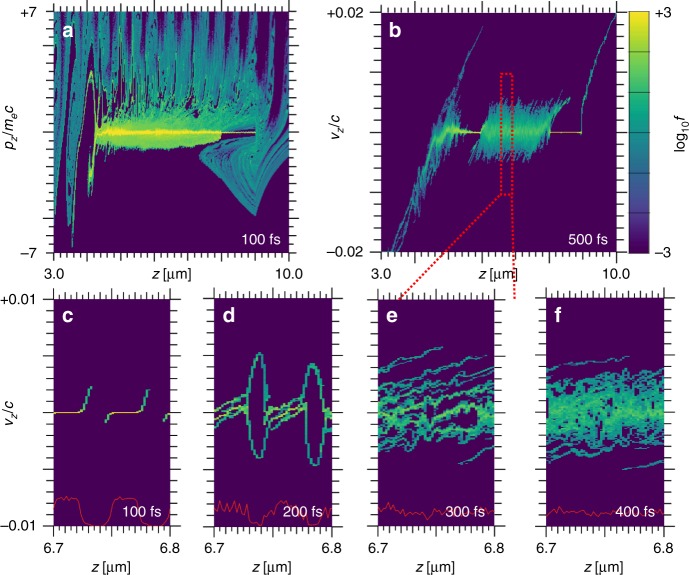
Details of the foam expansion. One-dimensional particle-in-cell simulation of an intense laser pulse irradiating an encapsulated nanofoam target. Laser comes from *z* = 0, foam located at 6–8 μm; shown are **a** electron velocity phase space at 100 fs; **b** ion velocity phase space at 500 fs; details of ion phase space in foam at **c**–**f** 100–400 fs

Interaction of the intense laser pulse with the preformed plasma generates hot electrons following a ponderomotive energy spectrum with a slope temperature $T_{{\mathrm{eff}}} = m_ec^2\left( {\sqrt {\left( 1 \right.} \left. { + a_0^2/2} \right) - 1} \right)\sim 1\,{\mathrm{MeV}}$^29^. Energetic electrons flood the target and cause rapid heating of background electrons in the foam elements. Figure 2 shows the time history of a laser interaction simulation in 1-D, which most clearly illustrates the physics. The electron phase space in Fig. 2a shows propagation of multi-MeV particles through the target and heating of the foam region. At first the electrons in the solid region behind the foam remain at a temperature <200 eV. Figure 2b shows the “snowplowed” ions from the front surface between *z* ≈ 4.5 µm and *z* ≈ 6.0^30^, the rapidly expanding foam ions between *z* ≈ 6µm and *z* ≈ 8.0 µm, and the rear surface TNSA accelerated ions at *z* > 9 μm.

Ions in the foam region get a double energy boost: first they accelerate when laser-generated MeV electrons arrive in the foam at ~100 fs and provide free charges in the vacuum gaps. Between 100–150 fs, the ions expand and reduce the electric field in the gaps so that ion acceleration stalls; the second boost occurs when the electron temperature in the filaments rises, around 150–200 fs, leading to enhanced charge separation fields. In particular, the ion velocity spectrum in the foam region at 400 fs is nearly uniform and non-directional in the foam region.

**Fig. 3:**
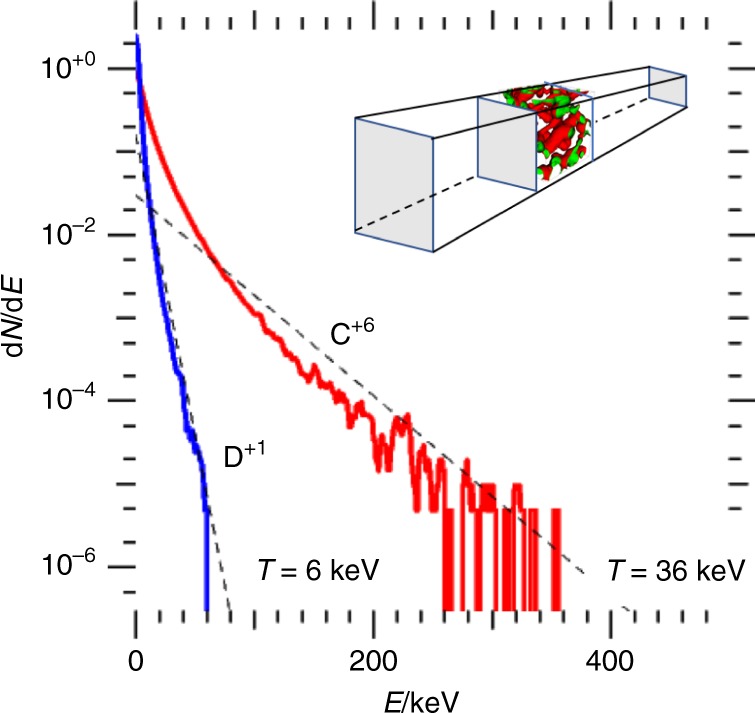
Result of a CD nanofoam explosion. Particle energy spectra of carbon (red) and deuterium (blue) ions from a 3-D particle-in-cell simulation of a laser-driven encapsulated CD nanofoam target, as illustrated in the inset. Dashed lines are exponential fits to the spectra

The ion energy spectrum resulting from a corresponding 3-D simulation (discussed below) is shown in Fig. 3. In a 1-D simulation there are no charge separation fields perpendicular to the laser propagation direction, however, the rise of the electron temperature in the filaments is isotropic. As a control we have modeled a solid target without any foam in 1-D. In this case, ion heating is caused mostly by resistive effects, i.e. by collisions of low-energy electrons with ions, yielding ion heating in solid density to less than 100 eV over a time interval of 500 fs; the electron-ion collisional heating time at solid density and an electron temperature of 1000 eV is several picoseconds. We have confirmed in additional simulations that our configuration is relatively insensitive to the thickness of the front or back solid layers; in fact, the main purpose of the back layer is to generate unambiguous measurements of accelerated ions. Our simulation gives a total coupling efficiency from the laser to electrons of about 40%; coupling from electrons to ions in the foam is about 5%, consistent with our fully integrated 3-D simulation described below. The overall coupling from laser to foam ions is about 1.5%.

### Scaling with foam density

The key target parameter that determines the ion energy spectrum is the average foam density. The average density *n*_*f*_ of a periodic arrangement of solid cylinders surrounded by vacuum is determined by the ratio of the gap between the cylinders, *L*_vac_, and the structural scale or period, *L* noted as, $A_s = \frac{{L_{{\mathrm{vac}}}}}{L}$. Assuming that the final particle number density *n*_*f*_ is uniform, this implies that $n_f = n_s(1 - A_s^2)$, where *n*_*s*_ is the intrinsic density of the solid foam ligaments. The peak kinetic energy an ion may obtain when electrostatically accelerated across a gap *L*_vac_ is *E*_*i*_ ~ |***E***|*L_vac_, where |***E***| is the peak field amplitude generated by the external source of hot electrons, i.e., by the laser intensity. At the same time, increasing the distance between filaments, while keeping their thickness *L*constant, reduces the final density *n*_*f*_. Increasing both the thickness of filaments and their spacing leads to an incomplete plasma expansion and to non-uniform density. In our case, we want to deplete the initial structure and obtain a uniform plasma density, so the filament thickness should be small compared to the rarefaction length *L* = *c*_*s*_*τ*_laser_, where τ_laser_ is the pulse duration and c_s_ is the sound speed. At the temperature observed in our 1-D PIC simulations, T_e_ = 20 keV, the sound speed is *c*_*s*_ = 10^−3^*c* which leads to an optimal structural size of *L* = 30 nm.

Assuming the foam ligament expansion results in a uniform density plasma given by $n_f = n_s(1 - A_s^2)$, one can calculate the aspect ratio *A*_*s*_ that maximizes the reaction rate $R\sim n_f^2 < \sigma v > \sim n_f^2T_i^\alpha$ using a value of *α* = 3 − 4, which is typical for light ion reactions in the temperature range of 10–30 keV. For example, using *α* = 3.5 results in $A_{s_{{\mathrm{opt}}}} \cong 0.5$. In a cylinder geometry this corresponds to a volumetric fill fraction of $\pi \left( {\frac{L}{4}} \right)^2/\left( {2L} \right)^2 \cong 0.2$, or a foam density that is 20% of the solid density of the ligaments. Since this result depends weakly on the actual value of *α*, it will change slightly with the target material and the nuclear reaction; the value used here serves as an example.

As mentioned above we can also tune the ion spectrum by changing the laser intensity. Using constant target parameters, we have confirmed with 3-D simulations like the one described below that the peak ion energy scales linearly with laser intensity between 10^19^–1020 W/cm^2^. This scaling holds because electron charge and energy currents are roughly proportional to intensity, while the absorption fraction remains relatively flat. For the nuclear reactions we are interested in, ion energies between 10–100 keV are most relevant; this motivates our choice of intensity.

### Integrated simulation of exploding foam target

Consider a foam that is made up of CD plastic fibers at solid density of 1.1 g/cc at a volume fill fraction of about 20% as motivated above, resulting in an average foam density of 0.23 g/cc. The initial foam structure is created from a series of three-dimensional random walks in a box with periodic boundary conditions, with a slowly varying thickness around an average of 25 nm; the initial density profile of each filament has a transverse scale of 4 nm to match the two-stage ion acceleration described above. Figure 1 shows a schematic view of the initial conditions with the inset showing the density contours of the 3D foam structure. Both orientation and thickness of filaments are randomly distributed in order to avoid anisotropies and periodic bunching in space that would taint measurements.

To verify ion energy distribution and isotropy we have performed fully integrated three-dimensional particle-in-cell simulations of a nanoscale structured plastic foam sandwiched between two thin solid density layers. The laser and plasma conditions are identical to the 1-D case discussed above. To ensure convergence at modest computational cost, and obtain multi-dimensional laser plasma interaction physics we used a transverse simulation box size of 1 µm, and box length of 12 µm. For comparison, typical focal spot sizes at the simulated intensities are between 5 and 50 microns in diameter. At a resolution of 240 cells per micrometer the total simulation size was 1.65 × 10^8^ cells and deployed roughly 3 × 10^9^ particles.

Figure 3 shows the resulting energy distribution of carbon and deuterium particles 400 fs after the onset of the initial laser energy. Both ion species have identical charge-to-mass ratio, q/m, and are present in equal numbers. Since the Vlasov-Maxwell equations scale with q/m, the ion velocity distribution functions of D^+^ and C^+6^ are identical—leaving collisional effects and particle statistics aside. The corresponding ion energy distributions scale as $\frac{{{\mathrm{d}}N}}{{{\mathrm{d}}E}} = \frac{{{\mathrm{d}}N}}{{{\mathrm{d}}v}} \ast \left( {\frac{{{\mathrm{d}}E}}{{{\mathrm{d}}v}}} \right)^{ - 1}\sim m_i^{ - 1}$ leading to a six times smaller peak ion energy for deuterium compared to carbon ions, which is confirmed in Fig. 3.

The energetic branch of the carbon spectrum, which contains about 30% of all particles above 10 keV, has a slope temperature of 36 keV. Simulations where deuterium ions are replaced by hydrogen yield similar ion spectra, however, since protons accelerate faster than deuterium ions, due to their smaller mass, they short out the accelerating field between filaments sooner. This leads to a slightly reduced peak energy for carbon ions when compared to CD foams of comparable density.

**Fig. 4:**
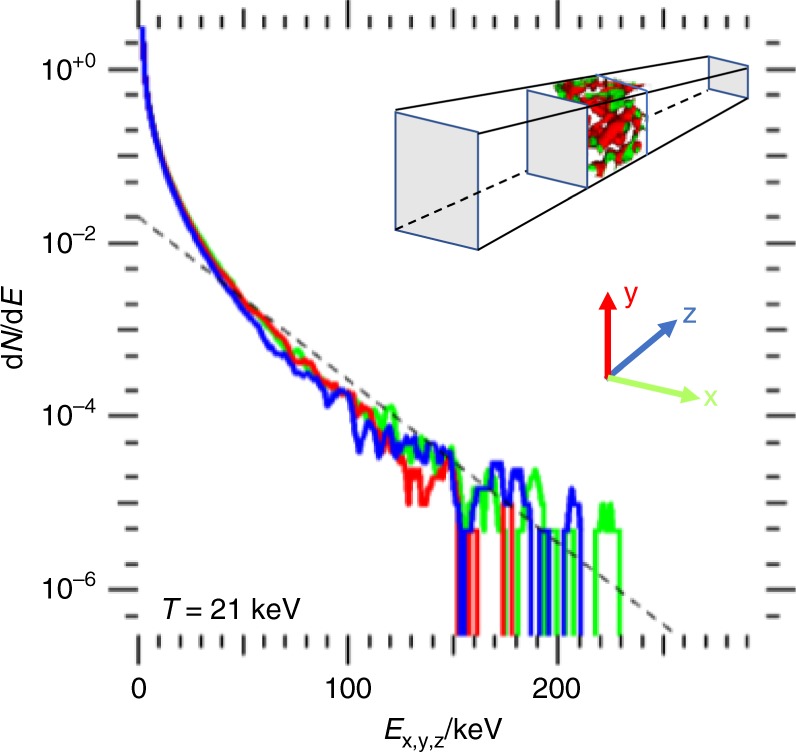
Isotropy of CD nanofoam explosions. Directional ion energy spectra in 3D particle-in-cell simulation, labeled by direction. Laser propagates along *z*-axis and is polarized along *y*-axis. Directional ion “temperatures” are reduced by a factor $\sqrt 3$ compared to the total; compare Fig. 3. (Dashed line represents an effective temperature for this distribution of 21 keV)

Figure 4 shows directionally-resolved energy spectra from our 3D simulation. Plotted is the number of particles per energy interval in the transverse (x, y) and longitudinal (z) directions. The directional energy is given by $E_x = M_ic^2[\sqrt {u_x^2 + 1} - 1]$ with *u*_*x*_ = *p*_*x*_/*M*_*i*_, for x, y and z, and *M*_*i*_ is the ion mass. The spectral densities are calculated using all ions in the foam region. The isotropy of the of energy spectra, which results from the amorphous, i.e., non-periodic, quasi-isotropic topology of the foam, makes this approach attractive for potential studies of nuclear processes in a plasma environment as it greatly simplifies characterizing the plasma conditions, and distinguishes this approach from that of others^11^ as discussed above. Collisional simulations on a smaller scale show that ion–ion collisions lead to a finite energy spread, but at a much slower rate than kinetic effects^31^.

To illustrate the importance of the foam surface isotropy, we have performed an additional 3-D simulation of a CD target made up from highly aligned nanowires (HANW) at the same volume fill fraction and electron distribution as in our reference case. The simulation results show a significant difference between ion spectra parallel and perpendicular to the axes of the nano-wires. This results from the reduced accelerating fields along the wire axes. In additional 2-D simulations, we have studied the effect of a finite size laser spot, or a finite foam volume. At a typical energy of 40 keV for Carbon ions, corresponding to a velocity of 0.3% of the speed of light, we observe a minimal impact on the uniformity of the heated foam region that can be mitigated by maximizing the laser spot size.

## Discussion

Generating distributions of ions with tens of keV energies at near solid density has the potential to enable measurement of astrophysically relevant nuclear reaction cross sections in plasma screened conditions, both of which have been historically difficult to access experimentally.

In accelerator experiments, where a low-density beam impinges on a cold solid target and reaction products, or their sequelae, are detected, measurement precision is limited by the decreasing probability of transmission through the Coulomb barrier of the reactant pair and the challenges presented in managing background suppression. For non-resonant charged particle induced reactions, the cross section for reactants *a* and *b*, is frequently written in the form, $\sigma = \frac{{S(E)}}{E}e^{ - \left( {E/E_G} \right)^{ - 1/2}}$, where *S*(*E*)is the astrophysical S-factor, comprising the nuclear physics of the reaction, and the exponential function, or Gamow Factor, with *E*_*G*_ ~ (*αZ*_*a*_*Z*_*b*_)^2^ determining the tunneling probability through the Coulomb barrier given the nuclear charges *Z*_*a*_, and *Z*_*b*_. At low energies the cross section *σ*(*E*) decreases strongly with center-of-mass energy *E* due to the rapidly decreasing Gamow factor. This is enhanced further with increasing nuclear charge. Thus, the reaction rates of mid- to high-*Z* materials are highly suppressed when compared to the rates of light ion species like the hydrogen isotopes. For example, at a temperature of 3.5 keV, typical of conditions at the National Ignition Facility^32^, the reactivities for D(d,n), or ^12^C(p, γ), are 2.8 × 10^−20^ cm^3^/*s*, and1.1 × 10^−33^ cm^3^/*s*, respectively, despite the S-factor for the latter reaction being roughly twice that of the former^33^.

Further, in the accelerator based approach, the nuclear charge is screened by the orbital electrons of the target ion, which is very different from that in a hot plasma, where free electrons screen the nuclear charge at Debye lengths which can approach that of the Bohr radius, in a stellar core. Models for the impact of plasma screening on the cross section have been developed^34^ and further tabulated^35^ for various reactions, but experimental validation and quantification have yet to be demonstrated and performed, and represent the focus of many laboratories around the world.

In order for the platform described in the above simulations to be of value and contribute useful experimental data, it will be necessary to demonstrate its ability to generate a significant number of nuclear reactions in a low background environment, using a modest scale facility, and with appropriate diagnostics. Below we discuss the status of these issues as we currently understand them, and conclude that we are not far from being able to make useful measurements of reactions, such as ^12^C(p, γ).

**Fig. 5:**
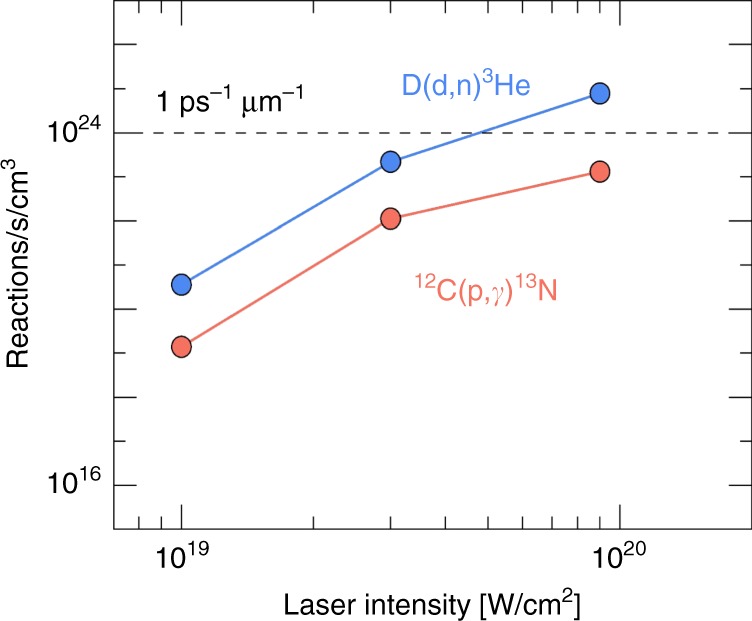
Sensitivity of nuclear reaction rate to changes in laser intensity. Nuclear reaction rates for ^12^C(p,γ)^13^N (lower curve) and D(d,n)^3^He (upper curve)

With regards to signal strength, Fig. 5 shows a comparison of reactivities between the carbon-hydrogen reaction ^12^C(p, γ)^13^N, which is relevant to the stellar CNO cycle, and the well-known D(d,n)^3^He reaction frequently used as a diagnostic in laser-generated high energy density science experiments. The reactivities are plotted as functions of laser intensity for 1 micron wavelength light. The ion energy spectra used to generate these plots were obtained from a series of three-dimensional PIC simulations like the one shown in Fig. 3, using CD and CH foams both with 1:1 atomic ratios, and at three laser intensities. The increase in intensity is linearly correlated with an increase in the slope temperature of the energetic ion component, hence the increase in reaction rate. We use the cross section data from standard nuclear data tables^33^ in the reference frame of one of the ions, typically the heavy ion when there are two different species. The D(d, n)^3^He reactivity climbs five orders of magnitude over this range of laser intensities. This rapid ascent of the reactivities with intensity is attributable to the increasing fraction of ions populating the high energy tails of the of ion energy distribution functions. Notably, the average total kinetic energies and ion cutoff energies remain below the thresholds for nuclear excitations in the ions, leaving elastic scattering and fusion as the only nuclear reactions occurring in these plasmas.

In addition to making reaction measurements feasible at energies below the capability of existing accelerator methods, the main differences between this approach and accelerator-based approaches are the ion beam current and exposure time. There are well-known limits to beam currents, requiring target exposure times to be sufficient to reach the high numbers of interacting ions relative to the integrated backgrounds. As an example, to get 10^22^ protons on a target requires 1600 Coulomb of beam charge. If the beam current is 1 mA, then 1.6 × 10^6^ s, or 18 days of operation is required. In our system this happens in a single shot. Further, Fig. 5 indicates that under the assumption of a 10^4^ µm^3^ volume, e.g. a 35 µm × 10 µm cylinder, that interacts for 1 ps, at an intensity approaching *I* ≈ 1 × 10^20^ W/cm^2^, roughly 10^4 12^C(p,γ) reactions are possible, as well as 10^6^ D(d,n) reactions. With proper experimental design, these represent a measurable number of reactions using the products as the primary diagnostics. While the number of reactions per individual shot in our approach is impressive, a fair comparison between the pulsed approach and the continuous one can be made applying the standard metric of luminosity ${\cal{L}}$; it is usually defined through the reaction rate

1 $$\frac{{{\mathrm{d}}R}}{{{\mathrm{d}}t}} = {\cal{L}}\sigma$$

where *σ* is a reaction cross section. In a traditional beam-target experiment, the luminosity ${\cal{L}}$ is defined as

2 $${\cal{L}} = {\mathrm{\Phi }}_{\mathrm{b}}\rho _T\ell _{{\mathrm{eff}}}$$

where *ϕ*_*b*_ is the flux or total number of particles in the beam per second and *ρ*_*t*_ is the target density, i.e., the number of field particles per volume, and $\ell _{{\mathrm{eff}}}$ is the effective target length. For an experiment like LUNA^36^, beam currents of order 500 *μ*A translate into a flux *Φ*_b_ = 3 × 10^15^ particles/Z/s where Z is the charge of the beam particle. A gas target is operated at low pressures to allow higher efficiency collection of the reaction products in the detector systems, typically 1 mbar, which sets the particle density of the target, *ρ*_*t*_ = *N*_*A*_*ρ*_*t*_/*A* ≈ 6 × 10^19^/*cm*^3^/*A* where *A* is the atomic mass of the target. The target length $\ell _{{\mathrm{eff}}} \approx 10\,{\mathrm{cm}}$. Thus, for LUNA we get ${\cal{L}} = 10^{35}\,{\mathrm{cm}}^{ - 2}\,{\mathrm{s}}^{ - 1}$. On the other hand, in our experiment where beam and target density coincide, the corresponding luminosity is ${\cal{L}} = v_{{\mathrm{ion}}}\rho ^2V$. With typical ion velocities of *v*_ion_ ≈ 10 ^8^cm/s and volumes *V* ≈ 1000 *μ*m^3^ in our approach, we get a per-shot luminosity of ${\cal{L}} \approx 3 \times 10^{44}A_1^{ - 1}A_2^{ - 1}\,{\mathrm{cm}}^{ - 2}{\mathrm{s}}^{ - 1}$, where *A*_*1,2*_ are the atomic numbers of the ion species involved in the reaction. However, since our system is pulsed with a life time of 500 fs per shot and a shot rate *f* per second, the time-averaged effective luminosity in our system is ${\cal{L}} = {\mathrm{f}} \times 10^{30}\,{\mathrm{cm}}^{ - 2}\,{\mathrm{s}}^{ - 1}$. Current repetitively fired short-pulse lasers operate with an f between 0.1 and 1^37^. Future high average power systems are being designed to operate at multi-kHz repetition rates, with *f* ≥ 1000^38^. In addition, their increased beam energies will support larger reacting volumes resulting in yet another factor of 10 increase in luminosity. Thus, in the coin of the accelerator realm, the conservative luminosity range of current and future systems extends from ${\cal{L}} = 10^{29}\,{\mathrm{cm}}^{ - 2}\,{\mathrm{s}}^{ - 1}$ to ${\cal{L}} = 10^{35}\,{\mathrm{cm}}^{ - 2}\,{\mathrm{s}}^{ - 1}$, which at the high end compares favorably with LUNA which is located deep underground in the Laboratori Nazionali del Gran Sasso^36^ to reject cosmic rays by a factor of 10^6^. The precise experimental timing and reactant entrance channel control of the proposed platform rejects background far more efficiently and could obviate the need for these remotely sited facilities in addition to producing statistically significant signal more efficiently. For example, a 1 Hz experiment, using time-of-flight detection is capable of cosmic ray rejection at a level of 1s/10 ns = 10^8^. Further, this platform enables experiments that may utilize direct product assay, e.g. ^13^N, and without contamination from alternative entrance channels, greatly enhance the efficiency of the experiment.

**Fig. 6:**
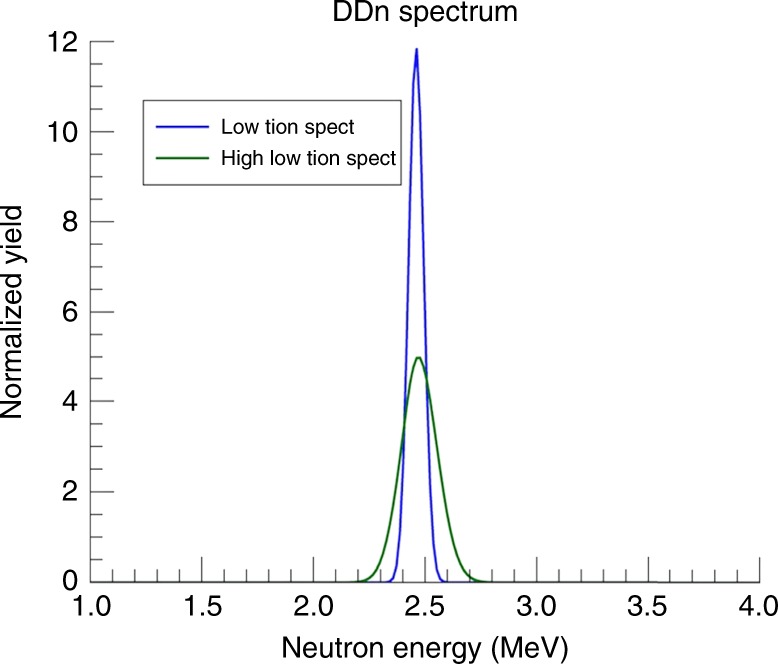
Distinctive neutron spectra help assess plasma conditions. D(d,n)^3^He reaction neutron energy spectra from single-temperature plasma (blue) and two-temperature ion distribution (green)

With regard to diagnosing the conditions and the signatures generated in such experiments, we note that the presence of a second ion temperature will alter the fusion neutron energy spectrum, produced in D(d,n) reactions, in measurable ways. For example, if foam targets are doped with a sufficient amount of deuterium, there will be a measurable number of 2.54 MeV fusion neutrons, whose energy spectrum may be sampled using spectroscopic techniques such as time-of-flight. Figure 6 shows a comparison between neutron spectra from a single-temperature plasma with one from a two-temperature plasma like that shown in Fig. 3 above. The distinct shape difference between these two spectra may be exploited to assess the conditions of the plasma. In the absence of large scale fluid dynamics, the spectral shape can be analyzed under the assumptions of a multi-temperature plasma with fit parameters that describe the fractions and temperatures of the various components. This approach can further be augmented with techniques, such as spectroscopic dopants, and X-ray Thomson scattering to assess the electron temperature and density of the plasma.

Another distinguishing feature of this platform is the distinct cutoff of the ion kinetic energy spectra and its dependence on the laser intensity. This cutoff further isolates these experiments from backgrounds due to other nuclear reactions, by allowing an experimenter to keep keep the ion distribution below the energy thresholds for the background reactions. Further, changing the laser intensity also allows the reactivity to be mapped out in energy space, the total reactions being the integral of the ion spectrum folded with the reactivity. A series of experiments done changing the laser intensity changes, and in turn, the high energy cutoff can be performed to map out the yield dependence on mean, or max energy of the distribution.

While inferring the plasma conditions from indirect diagnostics poses challenges, these experiments can be turned around to provide direct diagnostics using well-known reactions. In the example provided here of the ^12^C(p,g)^13^N reaction rate, a deuterium doped CH target would enable the use of the well-known D(d,n)^3^He reaction as a diagnostic. Studying the neutron yield requires isolating the 2.45 MeV neutron, and from that inferring the average kinetic energy of the deuterons as well as the total reacting volume and the duration. The known energy dependence of the cross section can be used to establish the high energy cutoff. Further, the co-loaded isotopes can be chosen to avoid producing backgrounds to the reactions of interest, and measure the same plasma in which those reactions are occurring. While this is not a “standard” way of producing cross section measurements for astrophysics, it is a straightforward extension of current methods used in high energy density science, and research in inertial confinement fusion.

Finally, rapidly varying cross sections provide a means of comparing the electron screening potentials obtained in cold-target experiments with the warm plasma conditions of this platform for known cross sections by comparing production rates.

Our platform has a number of attributes which make it suitable for measuring rare reaction rates. It creates solid density plasmas with high average kinetic energies in short times. The total yield for nuclear reactions depends on the square of the density of ions, allowing the experiments to probe “lower temperature” reactivities. The short duration is also an advantage by substantially reducing environmental backgrounds that are a dominant feature of traditional accelerator experiments.

In conclusion, we have proposed an intense short-pulse laser-based platform capable of producing nuclear reactions in micrometer scale, multi-keV, near-solid density plasmas. It is found that targets made from unstructured nanofoam tamped between solid layers and illuminated by an intense short-pulse laser, yield spatially homogenous, quasi-isotropic electron and ion distributions. Nuclear reactivities that are dominated by the Coulomb barrier will be drastically altered by the presence of non-thermal, multi-keV tails in the ion distribution. It appears that this platform can be developed to measure nuclear reactivities in an environment where plasma screening effects are important, unlike conventional measurements in accelerators. Deuterated foam targets will provide distinctly measurable neutron signals for ion temperature and density; the shape of the resulting DD neutron signal will allow one to deconvolve the ion distribution function in the foam. This ion distribution function can then be used to infer plasma conditions in the foam, to obtain reliable values for low reaction rates also present in the foam. This platform requires a high-contrast laser system, i.e. less than 1 mJ on target before the intensity reaches 10^18^ W/cm^2^ to prevent the foam from being heated by a shock, assuming a focal spot diameter of 100 um, while the laser pulse would contain 100 J of laser energy, consistent with the high repetition rate ELI-NP system. Even at 100 times reduced laser energy, i.e. at a focal spot diameter of 10 µm and 1 J, the interaction will produce an observable number of nuclear reactions; this has been shown in a recent experiment by one of us^22^.

## Methods

### Particle-in-cell simulations

The particle-in-cell simulation code PSC^25^ uses an explicit FDTD type Maxwell solver on a Yee mesh. We use a second order particle shape in order to avoid artificially softening the edges of the foam structure while avoiding numerical self-heating. The one-dimensional simulation shown in Fig. 2 uses a spatial resolution of 480 cells per micrometer and 14,400 electrons and ions combined; each cell contains an equal mixture of C^+6^ and D^+1^ ions, plus the corresponding number of electrons so that particle weight is uniform for all species. Our 3-D simulations, the results of which are shown in Figs. 3–5, have a reduced spatial resolution of 240 cells per micrometer and 18 (=4 + 14) ions and electrons per cell; we have verified numerical convergence in separate 3-D simulations. Our simulations use transversely periodic boundary conditions; this means that the laser is modeled as a plane wave, while introducing three-dimensional aspects of electron dynamics at the critical surface and for electron transport inside the foam structure.

## Data Availability

Simulation data that support the findings of this study are available on request from the corresponding author.
